# Feasibility of Repeated Patient-Reported Outcome Collection and Trial Design Implications for Structured Transition Care in Adolescents with Congenital Heart Disease: A Single-Center Pilot Randomized Controlled Study

**DOI:** 10.3390/children13060742

**Published:** 2026-05-26

**Authors:** Salvatore Angileri, Rosario Caruso, Serena Francesca Flocco, Irene Baroni, Gaia Spaziani, Silvia Favilli, Iacopo Olivotto, Daniele Ciofi, Ilaria Milani, Giulia Maga, Cristina Arrigoni, Arianna Magon, Maddalena De Maria

**Affiliations:** 1 Department of Biomedicine and Prevention, Tor Vergata University of Rome, via Montpellier 1, 00133 Rome, Italy; salvatore.angileri@meyer.it; 2 Operational Research Unit, Clinical and Cellular Physiopathology, Meyer Children’s Hospital IRCCS, 50139 Florence, Italy; 3 Department of Biomedical Sciences for Health, University of Milan, 20133 Milan, Italy; 4 Health Professions Research and Evidence Transfer Unit, IRCCS MultiMedica, 20099 Sesto San Giovanni, Italy; ilaria.milani@multimedica.it; 5 Health Professions Research and Development Unit, IRCCS Policlinico San Donato, 20097 San Donato Milanese, Italy; serena.flocco@grupposandonato.it (S.F.F.); irene.baroni@grupposandonato.it (I.B.); 6 Cardiology Unit, Meyer Children’s Hospital IRCCS, 50139 Florence, Italy; gaia.spaziani@meyer.it (G.S.); silviafavilli@gmail.com (S.F.); iacopo.olivotto@meyer.it (I.O.); 7 Department of Health Professions, Meyer Children’s Hospital IRCCS, 50139 Florence, Italy; daniele.ciofi@meyer.it; 8 Department of Public Health, Experimental and Forensic Medicine, University of Pavia, 27100 Pavia, Italy; giuliamaga01@gmail.com (G.M.); cristina.arrigoni@unipv.it (C.A.); arianna.magon@unipv.it (A.M.); 9 Department of Life Science, Health, and Health Professions, Link Campus University, 00165 Rome, Italy; m.demaria@unilink.it

**Keywords:** congenital heart disease, adolescence, transition care, pilot randomized controlled trial, patient-reported outcomes, feasibility, sample size planning

## Abstract

**Highlights:**

**What are the main findings?**

- Repeated collection of patient-reported outcomes in adolescents with congenital heart disease was feasible at 12 months (retention ≈ 61%), but follow-up completion was inconsistent across intermediate assessments, indicating that stable repeated measurements cannot be assumed across all time points.
- Although not powered for efficacy, the pilot provided preliminary evidence of potentially different longitudinal (ICC ≈ 0 vs. 0.56) and generated empirical estimates of missingness, variability, and sample size directly applicable to the design of a future confirmatory trial.

**What are the implications of the main findings?**

- A future confirmatory trial should prioritize retention at the final endpoint over uniform completion across all follow-ups, prespecify analytic strategies for incomplete repeated measurements, and incorporate structured retention-support mechanisms from the design stage.
- PCS12 at 12 months, analyzed cross-sectionally with baseline adjustment, may warrant further evaluation as one possible endpoint option in a future confirmatory trial; a baseline-adjusted cross-sectional approach was associated with a lower estimated sample size than a change-score formulation in the observed data, though this observation should be regarded as an exploratory planning signal only.

**Abstract:**

**Background/Objectives**: Structured transition care models for adolescents with congenital heart disease (CHD) are increasingly advocated, but methodological evidence to support the design of adequately powered randomized trials remains limited. This pilot randomized study was designed primarily to assess the feasibility of repeated patient-reported outcome (PRO) collection and to generate empirical parameters for planning a future confirmatory trial, rather than to formally evaluate intervention efficacy. **Methods**: This was a single-center, parallel-group, pilot randomized controlled trial conducted at Meyer Children’s Hospital, Florence, Italy, within the TELEMACO project (NCT05713591). Twenty-three adolescents with CHD were randomized 1:1 to a structured transition care intervention (n = 11) or usual care (n = 12). PROs, including the SF-12 Physical (PCS12) and Mental (MCS12) Component Summaries, health engagement, life satisfaction, and healthcare needs, were collected at baseline and at 3, 6, 9, and 12 months. Pre-specified exploratory analyses addressed retention, missingness, linear mixed-effects models, intraclass correlation coefficients (ICCs), and sample size scenarios. **Results**: Retention at 12 months was 63.6% (intervention) and 58.3% (control), with substantially lower completion rates at intermediate assessments (T2–T3: 27–50%), directly affecting the reliability of longitudinal estimates at those time points. Mixed-effects models showed no significant time-by-group interaction for PCS12 (*p* = 0.13) or MCS12 (*p* = 0.39); unadjusted contrasts suggested nominally higher PCS12 values in the intervention group at selected assessments. ICCs were approximately 0 for PCS12 and 0.56 for MCS12, indicating fundamentally different variance structures. **Conclusions**: Repeated PRO collection was feasible, though retention across intermediate assessments was inconsistent. The pilot generated empirically grounded estimates for the design of a future confirmatory trial. Sample-size scenarios were highly sensitive to uncertainty in the PCS12 variability estimate, ranging from approximately 25 to 115 analyzable participants per group, depending on the true standard deviation. Within this pilot dataset, PCS12 at 12 months, analyzed cross-sectionally with baseline adjustment, emerged as a provisional endpoint option requiring further evaluation in an adequately powered confirmatory trial.

## 1. Introduction

Congenital heart disease (CHD) is among the most prevalent structural birth defects, affecting approximately 8–9 per 1000 live births, and improvements in surgical and medical care have led to a substantial increase in survival into adulthood [1,2]. As a result, the population of adolescents and young adults living with CHD is growing, and the transition from pediatric to adult-oriented care has become a critical phase in long-term disease management [3]. During this period, patients are expected to progressively assume responsibility for their health, develop adequate disease knowledge, maintain adherence to follow-up recommendations, and engage in self-management behaviors, while simultaneously navigating the broader psychosocial challenges of adolescence, including identity formation, autonomy development, and future planning [4,5,6].

Suboptimal transition has been associated with loss to follow-up, reduced adherence, and potentially preventable adverse outcomes, including unplanned hospitalizations and worsening clinical status [6,7,8]. To address these challenges, structured transition care models have been proposed and implemented in several centers, generally combining standardized education, multidisciplinary counseling, and coordinated support across the transition process [7,9,10,11]. Early evidence suggests that such interventions may improve patient-reported outcomes, including disease knowledge, health engagement, adherence, and health-related quality of life (HRQoL) [10,11,12,13,14,15,16,17,18,19]. However, the available evidence suggests that additional randomized controlled trials are needed to strengthen the evidence in this population supporting structured transition care models [19].

Accordingly, beyond the question of intervention effectiveness, the field still lacks key methodological information needed to plan adequately powered confirmatory trials [20,21,22]. In particular, important design parameters remain poorly characterized in real-world CHD transition settings, including retention across repeated follow-up assessments, the extent and structure of missing patient-reported outcome (PRO) data, the plausibility of missing-at-random assumptions, the within-subject stability of HRQoL measures over time, and the variance components required for sample size estimation [10,20,21]. This uncertainty is especially relevant in pediatric and adolescent longitudinal research, where attrition, intermittent missingness, and variability in follow-up completion are common and may substantially affect the interpretability of results [9].

In this context, pilot randomized studies could play an important methodological role in addressing these uncertainties [23]. When designed to inform future trial planning rather than to formally test efficacy, they inform researchers by providing empirical estimates of retention, characterizing missingness patterns, assessing whether longitudinal modeling approaches that accommodate incomplete follow-up are plausible, quantifying within-subject correlations and intraclass correlation coefficients for key outcomes, and supporting data-driven sample size scenarios for subsequent trials [23,24,25]. Such information is important for determining whether a definitive randomized study is feasible, which analytic strategy is most appropriate, and which design assumptions should guide future planning.

The present study reports a single-center pilot randomized controlled trial conducted in adolescents with CHD within the context of a structured transition care intervention. Rather than aiming to formally evaluate intervention efficacy, this pilot was designed to generate empirical methodological information to support the planning of a future adequately powered randomized controlled trial. Specifically, the study aimed to evaluate the feasibility of repeated PRO collection across multiple follow-up assessments; quantify participant retention over time and by randomized group; examine the extent, distribution, and structure of missing data; and describe longitudinal patterns of patient-reported outcomes in the intervention and control groups. In addition, exploratory linear mixed-effects models were used to examine whether outcome trajectories differed between groups over time while accounting for within-subject correlations and incomplete follow-up. Intraclass correlation coefficients and observed variability estimates were derived to inform sample size planning for a future confirmatory trial.

## 2. Materials and Methods

### 2.1. Design

This was a single-center, parallel-group, pilot randomized controlled trial conducted at Meyer Children’s Hospital, Florence, Italy, reported following “Consolidated Standards of Reporting Trials” (CONSORT) principles [25]. The study was designed to: (i) evaluate the feasibility of repeated PRO collection across multiple follow-up assessments; (ii) quantify participant retention over time and between randomized groups; (iii) characterize the extent, distribution, and structure of missing data and explore the plausibility of the missing-at-random assumption; and (iv) provide empirical estimates of longitudinal outcome variability, within-subject correlation, and intraclass correlation coefficients for key PRO measures.

This study was conducted within the broader framework of the TELEMACO project (ClinicalTrials.gov ID: NCT05713591), a multicenter randomized study evaluating a structured transition care model for adolescents with CHD. The present manuscript reports the Meyer site experience as a standalone pilot analysis, with the aim of providing a focused methodological and descriptive evaluation of longitudinal data completeness, outcome trajectories over follow-up, and design parameters informative for a future confirmatory trial.

Participants were randomly allocated in a 1:1 ratio to either a structured transition care intervention or usual care. Outcome assessments were performed at baseline (T0) and at four pre-specified follow-up time points: 3 months (T1), 6 months (T2), 9 months (T3), and 12 months (T4). At each assessment, patient-reported outcomes were collected, encompassing health-related quality of life (HRQoL), healthcare needs, health engagement, and self-rated life satisfaction. Baseline socio-demographic and clinical characteristics were recorded at study entry (see Section 2.4).

Consistent with the pilot and feasibility nature of the study, all outcome analyses were pre-specified as exploratory [24,26]. The study was not powered to conduct confirmatory hypothesis testing of effectiveness outcomes. Accordingly, analytical emphasis was placed on feasibility indicators, descriptive longitudinal summaries, exploratory linear mixed-effects model analyses, and the estimation of design parameters for a future confirmatory trial.

### 2.2. Setting, Participants, and Sample Size

The study was conducted at Meyer Children’s Hospital, Florence, Italy, a tertiary pediatric referral center with a dedicated pediatric cardiology and CHD follow-up program. Data collection was conducted between January 2022 and June 2025. All participants were enrolled and followed within this single-center setting; the present report is restricted to participants enrolled at the Meyer site and does not include data from other TELEMACO centers.

Eligible participants were adolescents with a confirmed diagnosis of CHD who were undergoing active follow-up in the pediatric cardiology service and were considered appropriate candidates for transition-oriented care. Inclusion criteria were: diagnosis of moderate or complex CHD documented in the clinical record; age within the adolescent range defined by the parent TELEMACO protocol (12–18 years); active follow-up at the Meyer pediatric cardiology service; ability to understand and complete self-administered patient-reported outcome questionnaires in Italian; and written informed consent from parents or legal guardians, with participant assent where applicable. Exclusion criteria were: major cognitive impairment or neurodevelopmental condition precluding valid questionnaire completion; severe clinical instability preventing participation in the transition pathway; inability to participate in scheduled follow-up assessments; and any condition judged by the clinical team to make participation inappropriate or not meaningful.

The sample size for this pilot study was not determined through formal power calculations for effectiveness outcomes, consistent with the methodological guidance for pilot and feasibility randomized trials [24,26]. Rather, the enrolled sample was sized to generate empirical estimates of the parameters required as inputs for the design of a future confirmatory trial, specifically: participant retention across five longitudinal assessment points; the extent and structure of missing PRO data; within-subject outcome variability; and intraclass correlation coefficients for the primary HRQoL measures. These parameters cannot be reliably assumed from the existing literature in this population. The final pilot sample comprised 23 participants, randomized to the intervention (n = 11) and control (n = 12) groups, and followed longitudinally from baseline to 12 months across five assessment time points (T0–T4).

### 2.3. Intervention and Usual Care

Participants allocated to the intervention arm received a structured transition care intervention derived from the Italian transition model previously developed and preliminarily evaluated in adolescents with CHD [13,16]. In operational terms, the intervention was delivered over the 12-month follow-up period through a combination of scheduled transition encounters and needs-based contacts. The core pathway included a structured educational session, repeated counseling/support contacts, and coordinator-led transition reviews aligned with the planned study assessments. Sessions were delivered primarily by a specialized nurse acting as transition coordinator, in collaboration with the pediatric cardiology team and, when needed, other professionals involved in psychosocial support. Educational and counseling contacts were delivered face-to-face during scheduled clinical or study visits, with additional telephone-based contacts when required to support continuity and address individual needs. The duration of each contact was adapted to participants’ needs and clinical scheduling constraints, and intervention exposure was documented in clinical session records. Educational sessions lasted approximately 50 min, while counseling/coordinator contacts lasted approximately 45 min. This model was conceived as a standardized yet personalized transition pathway to support adolescents as they progress from pediatric-centered care to more autonomous, adult-oriented management of their condition. Within this framework [13,16], transition was not intended as a single transfer event, but as a longitudinal care process combining education, psychosocial support, and coordination of care according to the developmental and clinical needs of the adolescent [27].

The transition model was built around three core components [13,16]. The first component focused on improving understanding of the clinical condition through tailored education. Adolescents received disease-specific information adapted to their congenital heart defect phenotype and clinical history, delivered through a structured educational package that included verbal explanations, printed educational materials, and face-to-face discussions with healthcare professionals. Educational content addressed the nature of the heart defect, prior surgical or corrective procedures, symptoms, pharmacological treatments, and the rationale for long-term cardiac follow-up. In addition, the educational pathway covered practical lifestyle and developmental topics that are particularly relevant during adolescence and transition, including physical activity, nutrition, oral hygiene, tattoos and piercings, vaccination, contraception, and pregnancy-related issues. In the original Italian model, this educational component was considered essential for promoting knowledge of disease and fostering progressive responsibility for one’s own care.

The second component focused on psychological and coping support. The Italian transition model had been previously conceptualized as including structured opportunities for discussion and counseling aimed at helping adolescents and their families deal with the emotional and practical challenges associated with CHD during transition. These moments were intended to facilitate the development of functional coping strategies, reinforce motivation, and support a more active engagement with care. In the original model, this component could involve counseling interactions with trained professionals and discussions centered on both the lived experience of illness and the educational content introduced during the transition pathway. The underlying rationale was that improving disease knowledge alone would be insufficient unless accompanied by support for the adolescent’s emotional adjustment, self-confidence, and capacity to manage uncertainty and future planning.

The third component focused on engagement and coordination of care. In the Italian model, transition was coordinated by a dedicated transition coordinator, typically a specialized nurse, who acted as the organizational and relational hub of the pathway. This role included coordinating the timing and delivery of the educational and counseling components, reviewing the achievement of transition goals, identifying unmet needs, and facilitating communication among adolescents, families, and the multidisciplinary team. The coordinator also had the function of adapting the pathway to individual needs, including through additional in-person or telephone-based contacts when needed. This component reflected the broader conceptualization of transition as a coordinated developmental process rather than a single clinical handover.

Intervention delivery was monitored by the transition coordinator throughout the study period. Adherence to the transition pathway was tracked using clinical session records, documenting each participant’s exposure to the three core intervention components. Regarding the educational component, 9 of 11 intervention participants completed the full structured educational package; the remaining two participants received a partial educational session due to clinical scheduling constraints unrelated to study procedures. Counseling contacts were documented for all 11 participants, with a median of 4 sessions per participant (IQR 3–5) over the 12-month follow-up period. Coordinator interactions occurred at all scheduled assessment time points for 8 of 11 participants; the remaining three participants missed at least one coordinator contact, attributable to logistical factors such as school commitments or rescheduled clinical appointments. Overall, the intervention was delivered with acceptable consistency across participants; minor variations in exposure were attributable to logistical factors inherent to a pragmatic single-center pilot and are acknowledged as a potential source of heterogeneity in intervention dose.

Participants allocated to the control arm received usual care. Usual care consisted of routine clinical follow-up without the structured and standardized transition pathway described above. In particular, patients in the control group did not receive the formalized educational package, the predefined counseling moments, or the coordinated transition activities characterizing the intervention model, although they continued to receive standard cardiology follow-up according to local clinical practice. Outcome data were collected in both groups at the same scheduled assessment time points.

### 2.4. Outcomes and Data Collection

Data collection was structured around one baseline assessment and four longitudinal follow-up assessments over a 12-month period. Baseline assessment (T0) was performed at study entry and included socio-demographic and clinical characteristics, as well as PROs. Follow-up assessments were scheduled at 3 months (T1), 6 months (T2), 9 months (T3), and 12 months (T4) after baseline. According to the parent TELEMACO protocol, follow-up outcome collection was planned at 3-month intervals over 1 year in both study groups to describe changes in patient-reported outcomes over time. The 3-month interval was selected to capture the temporal dynamics of the transition process across its key developmental and clinical phases, to provide a sufficient number of repeated observations for longitudinal modeling, and to align with assessment schedules used in comparable CHD transition research [13]. However, this frequency implied five assessment occasions over 12 months, which may have represented a meaningful burden for adolescent participants alongside routine clinical follow-up and other age-related commitments. The extent to which this schedule contributed to intermittent non-response at intermediate assessments is acknowledged as a relevant consideration for the design of the future confirmatory trial.

All patient-reported outcomes were completed directly by participants via self-administered questionnaires at each scheduled assessment time point. Data collection was coordinated by the study transition coordinator, who distributed the questionnaires, provided assistance with comprehension if requested, and ensured completeness of responses, but did not rate or score outcomes independently. Given the self-report nature of all outcome measures, formal assessor blinding was not applicable; however, as participants were aware of their group allocation, the potential for response bias cannot be excluded and is acknowledged as a limitation of the study design.

Baseline socio-demographic and clinical variables included age, sex, marital status, education, employment status, body mass index, diagnostic category, comorbidity burden, and New York Heart Association (NYHA) functional class, as available in the study dataset [28]. These variables were used to describe the pilot sample at study entry and to explore whether missing follow-up outcome data were associated with baseline participant characteristics.

The main longitudinal outcomes were PROs collected repeatedly across study visits. Health-related quality of life was assessed using the 12-Item Short Form Health Survey (SF-12), from which the Physical Component Summary (PCS12) and Mental Component Summary (MCS12) scores were derived [15,29]. These two measures were considered the main quality-of-life outcomes and were central to the exploratory longitudinal analyses and to the estimation of variance components for future trial planning.

Additional validated PROs included health engagement, measured with the Patient Health Engagement (PHE) Scale [30]; self-rated life satisfaction, assessed through a visual analogue scale (VAS) [31]; and healthcare needs, assessed as perceived by adolescents using the validated Italian version of the Healthcare Needs Scale for Youth with Congenital Heart Disease (I-HNS-CHD-s), summarized as both an overall score and domain-specific scores reflecting healthcare education, clinical support, emotional support, and continuum of care [32]. These outcomes were included to provide a broader description of patient-reported status over the follow-up period and to support an exploratory assessment of data completeness across measures.

Given the methodological purpose of the pilot, outcome collection was evaluated not only by observed scores but also by feasibility indicators. For this reason, repeated outcome data were used to quantify retention at each follow-up time point, describe the extent and distribution of missing data across outcomes and assessments, and explore the structure of incomplete follow-up. A participant was considered retained at a given follow-up assessment if at least one PRO was available at that time point. This operational definition was adopted to capture effective participation in longitudinal outcome collection in a pragmatic manner consistent with the pilot design. For the purpose of informing sample size planning for the future confirmatory trial, a second and more stringent metric was also derived: the proportion of randomized participants with a complete set of primary outcome data, defined as the availability of both PCS12 and MCS12 scores from the SF-12, at the final follow-up assessment (T4). This metric, referred to hereafter as primary outcome completion, is distinguished from the broader retention definition and represents the most directly relevant parameter for attrition adjustment in future trial design, as it reflects the proportion of participants from whom the candidate primary endpoint could actually be derived.

To support planning for a future confirmatory trial, the study also derived methodological parameters from repeated outcome data, including within-subject correlations across time points, intraclass correlation coefficients for the primary quality-of-life outcomes, and empirical estimates of outcome variability at the final follow-up and in the baseline-to-final change. These parameters were used to inform preliminary sample size scenarios for a future randomized controlled trial.

#### Safety and Adverse Events

Given the non-pharmacological and educational-supportive nature of the intervention, no intervention-related physical adverse events were anticipated. During follow-up, clinically relevant concerns arising during transition encounters or routine cardiology visits were managed according to local clinical practice and documented in the clinical record. No intervention-related adverse events or serious adverse events were reported during the pilot study. However, adverse events were not collected using a dedicated standardized harms form, and this is acknowledged as a limitation of the pilot design. In the future confirmatory trial, adverse-event and unintended-effect monitoring will be formalized prospectively, including potential psychological distress, excessive burden related to repeated assessments, and any clinically relevant concerns emerging during transition contacts.

### 2.5. Randomization and Allocation Concealment

Participants were randomly allocated in a 1:1 ratio to either the structured transition care intervention or usual care. Randomization was performed using a pre-specified, center-specific randomization list generated prior to study initiation, based on permuted blocks of size 4 to ensure approximate balance between groups throughout enrolment. Group assignment was determined by the participant’s chronological order of enrolment at the Meyer site, with each consecutive participant assigned to the group corresponding to their position in the pre-determined allocation list.

Group assignment was determined from a pre-specified, sealed, center-specific randomization list, applied sequentially following confirmation of eligibility and written informed consent. However, because assignments were determined by the participant’s sequential position in a permuted-block list with a block size of 4, the enrolling investigator could potentially anticipate the next group assignment, particularly toward the end of each block, which represents an important limitation in allocation concealment. This may have introduced a risk of selection bias, whereby awareness of upcoming assignments could have influenced eligibility assessment or the timing of enrollment decisions. Randomization and baseline assessment were performed at study entry, prior to the start of the allocated care pathway.

As this was a pragmatic pilot study conducted in a real-world clinical setting, allocation was not managed through a centralized randomization system. The absence of central concealment is a methodological limitation of this pilot and will be addressed in the design of the future confirmatory trial by implementing a web-based centralized randomization platform.

### 2.6. Blinding

Blinding was not implemented for participants or care providers. Given the nature of the intervention, which consisted of a structured transition care pathway including education, counseling, and coordinated follow-up activities, masking of treatment allocation was not feasible in practice. Participants and clinicians were therefore aware of group assignment throughout the study.

The statistician conducting the analyses was blinded to treatment allocation. Outcome assessments were performed by participants themselves through self-administered questionnaires, coordinated by the study transition coordinator, who was aware of group allocation. Because all primary and secondary outcomes were patient-reported measures, formal assessor blinding in the conventional sense was not applicable; nonetheless, participant-level blinding was not possible given the nature of the intervention, and the potential for response bias due to awareness of group allocation cannot be excluded. The statistician conducting the primary analyses remained blinded to treatment allocation throughout the analysis phase to minimize the risk of analytical bias. Given the pilot and exploratory purpose of the study, the use of a blinded statistical analysis was intended to reduce the risk of analytical bias, while the overall emphasis of the study remained on descriptive and methodological objectives rather than confirmatory inference on treatment efficacy.

### 2.7. Statistical Analysis

All analyses were conducted with a primarily exploratory aim, consistent with the pilot and feasibility nature of the study [23,26]. The study was not powered for confirmatory hypothesis testing of intervention effectiveness; accordingly, emphasis was placed on describing feasibility indicators, characterizing missing data and retention, conducting exploratory longitudinal modeling, and estimating parameters relevant to planning a future confirmatory trial.

Categorical variables were summarized as counts and percentages; continuous variables were described using medians and interquartile ranges, given the small sample size and the exploratory nature of the analyses. Baseline characteristics were summarized separately for the intervention and control groups without formal hypothesis testing, as inferential comparisons of baseline characteristics are not appropriate in randomized studies and were not pre-specified.

Retention was evaluated at each scheduled assessment time point (T0–T4) and by randomized group. A participant was considered retained at a given time point if at least one PRO measure was available at that assessment. Retention rates were calculated using the total number of randomized participants in each group as the denominator and were intended to provide empirical estimates of follow-up completeness to inform attrition assumptions for future trial planning [33]. Separately, primary outcome completion at T4, defined as the availability of both PCS12 and MCS12 from the SF-12 at the final assessment, was calculated overall and by randomized group, and used as the attrition parameter in sample size planning scenarios reported in Section 3.6.

Missing data were examined descriptively across variables, time points, and randomized groups. Overall proportions of missing values were calculated for each study variable and further stratified by assessment time point and treatment group. Patterns of missingness were explored to distinguish isolated missing values from broader patterns of incomplete follow-up. An individual-level binary indicator of missing longitudinal PRO data was constructed to assess whether participants with any missing outcome data differed from those with complete follow-up in baseline characteristics, including baseline PCS12 and MCS12 scores; these comparisons were performed using nonparametric methods (Wilcoxon rank-sum test and chi-square test). To further examine the plausibility of the missing-at-random assumption, an exploratory logistic regression model was fitted with a binary indicator of complete outcome missingness at each time point as the dependent variable and assessment time, randomized group, and their interaction as predictors [34]. Given the small sample size, the convergence and numerical stability of this model were evaluated, and the results were interpreted descriptively rather than inferentially. In the event of complete or quasi-complete separation, i.e., an anticipated risk in small, sparse datasets with rare events, logistic regression was to be deemed not feasible and replaced by a descriptive comparison of missing proportions between randomized groups at each time point.

Longitudinal PROs were summarized descriptively at each time point within each randomized group and reported as median and interquartile range. Exploratory between-group comparisons at individual time points were performed using Mann–Whitney U tests and are reported descriptively only. No adjustment for multiple comparisons was applied, and these analyses were not interpreted as confirmatory.

To explore whether longitudinal outcome trajectories differed between groups over time, linear mixed-effects models were fitted for the two primary HRQoL outcomes, PCS12 and MCS12. Fixed effects included assessment time (modeled as a categorical factor), randomized group, and the time-by-group interaction. A participant-specific random intercept was included to account for the within-subject correlation arising from repeated measurements. This specification corresponds to a compound symmetry marginal covariance structure, which assumes equal within-subject correlations across all pairs of time points and equal residual variances. This parsimonious parameterization was preferred over more complex structures, such as unstructured or autoregressive AR(1), given the small sample size and the substantial risk of model overparameterization with n = 23 participants and up to five repeated time points per individual. Models were estimated using restricted maximum likelihood (REML) for variance component estimation. These models were primarily intended to explore longitudinal patterns while accommodating incomplete follow-up under a missing-at-random framework, and to provide variance estimates and hypothesis-generating signals rather than definitive evidence of treatment effects. Model-based estimated marginal means and exploratory between-group contrasts at each time point were derived. The adequacy of model assumptions was evaluated through visual inspection of residual plots and normal Q-Q plots.

For descriptive and methodological purposes, within-subject correlations of PCS12 and MCS12 across follow-up time points were estimated using pairwise complete-case analyses, and correlation matrices were reported along with the number of paired observations contributing to each coefficient. Intraclass correlation coefficients (ICCs) for PCS12 and MCS12 were derived from the variance components of the mixed-effects models as the ratio of between-subject variance to total variance, and were used to characterize the relative contribution of between-subject and within-subject variability in the two primary HRQoL outcomes.

To support planning of a future confirmatory randomized controlled trial, empirical variability parameters were derived from the pilot dataset, including the standard deviation of PCS12 at the final follow-up assessment (T4), the standard deviation of individual baseline-to-T4 change scores, ICC estimates for the primary outcomes, and the observed retention rate at T4. These parameters were used to construct pragmatic sample size scenarios under two alternative endpoint definitions, i.e., a cross-sectional endpoint at T4 and a change-score approach, assuming a two-sided α = 0.05 and 80% power, with inflation for observed attrition. Given the small number of participants with available PCS12 data at T4, uncertainty around the observed standard deviation was quantified using a 95% confidence interval for the true standard deviation based on the chi-square distribution. Sensitivity sample size scenarios were then calculated using the lower bound, point estimate, and upper bound of this interval, while retaining the same assumed between-group difference, alpha level, power, and attrition inflation. All estimates were interpreted as provisional and intended to inform planning rather than to provide definitive design constants. All analyses were performed using R version 4.5.0 (R Core Team, 2025).

## 3. Results

### 3.1. Participant Flow, Sample, and Baseline Characteristics

**Figure 1:**
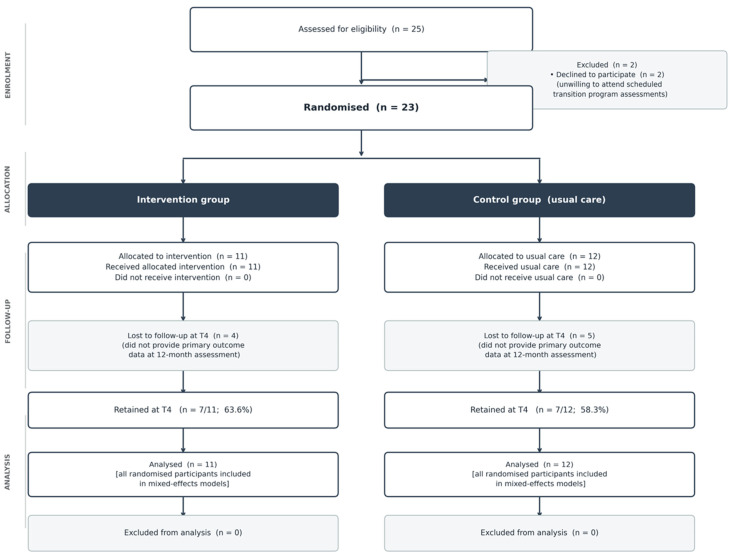
CONSORT 2025 Flow-diagram.

A total of 25 adolescents were assessed for eligibility. Of these, 2 declined to participate because they were unwilling to attend scheduled transition program assessments, and 23 were randomized. Participants were allocated in a 1:1 ratio to the intervention group (n = 11) or the control group receiving usual care (n = 12). All randomized participants received the allocated condition (Figure 1).

At the final follow-up assessment (T4, 12 months), 7 of 11 participants in the intervention group (63.6%) and 7 of 12 participants in the control group (58.3%) provided primary outcome data. Accordingly, 4 participants in the intervention group and 5 in the control group were considered lost to follow-up for the primary endpoint at T4. Despite incomplete follow-up at the final assessment, all randomized participants contributed available data to the longitudinal mixed-effects analyses, and no participant was excluded from the main exploratory longitudinal models.

Baseline characteristics of the randomized sample are shown in Table 1. The two study groups were broadly comparable with respect to age, education, body mass index, and baseline patient-reported outcomes, although some imbalances were observed, as expected in a small pilot randomized study. In particular, the control group included a higher proportion of female participants, whereas the intervention group showed slightly higher baseline MCS12 and healthcare needs scores. No baseline data were missing. These summaries are presented descriptively and are not intended for confirmatory between-group inference.

### 3.2. Feasibility of Repeated Outcome Collection: Retention and Missing Data

**Figure 2:**
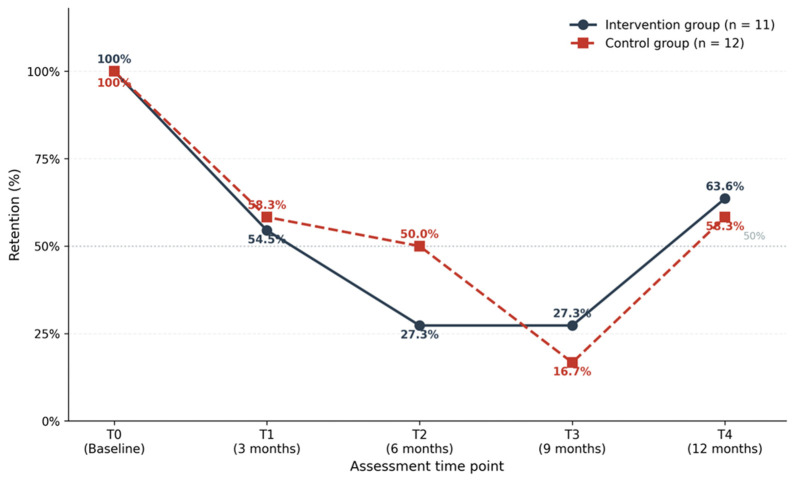
Retention across follow-up assessments by randomized group.

Retention over follow-up is shown in Figure 2. In the intervention group, retention was 100% at T0, 54.5% at T1, 27.3% at T2, 27.3% at T3, and 63.6% at T4. In the control group, corresponding values were 100%, 58.3%, 50.0%, 16.7%, and 58.3%, respectively. When applying the more stringent definition of primary outcome completion, availability of both PCS12 and MCS12 at T4, 7 of 11 participants in the intervention group (63.6%) and 7 of 12 in the control group (58.3%) had complete primary outcome data at the final assessment, corresponding to 14 of 23 randomized participants overall (60.9%). In this dataset, primary outcome completion and broad retention were numerically equivalent at T4, as all participants considered retained at that time point had completed the SF-12 in full. The distinction between the two definitions is nonetheless reported explicitly, as it may not hold in future trials where primary and secondary outcomes are collected via separate instruments. These figures indicate that the pilot demonstrated the feasibility of obtaining partial follow-up data at the final assessment rather than stable repeated measurements across all planned time points. Completion rates at intermediate assessments, particularly at T2 (27.3% and 50.0% in the intervention and control groups, respectively) and T3 (27.3% and 16.7%), were substantially lower than at T4, and this pattern directly limits the reliability of longitudinal estimates derived from intermediate observations, including correlation coefficients and mixed-effects model parameters at those time points.

Missing data were limited at baseline and were concentrated in longitudinal PRO measures. No missing values were observed for baseline socio-demographic and clinical variables. Across all study variables, the highest proportion of missing data was observed for health engagement (13 missing values, 20.3%), whereas missingness for the other repeated outcomes was lower: 6.3% for life satisfaction VAS and the continuum-of-care domain, 4.7% for the overall healthcare needs score, and for the clinical support, emotional support, and education domains. All remaining variables had no missing values.

Missingness increased over time, consistent with the decline in follow-up completion across repeated assessments, but did not appear to differ meaningfully between randomized groups. Exploratory comparisons based on an individual-level indicator of any missing longitudinal PRO data showed no evidence that participants with incomplete follow-up differed from those with complete follow-up in baseline PCS12 or MCS12 scores. In addition, the proportion of participants with any missing PRO data was similar across the intervention and control groups, not identifying obvious differential missingness between groups; however, these analyses do not provide meaningful support for the missing-at-random assumption, which was adopted as a working analytical framework only. Baseline comparisons alone cannot verify missing-at-random, because they do not exclude dependence of later missingness on intermediate PRO values or on external variables not captured in the dataset.

The pre-specified logistic regression model could not be estimated in a meaningful way due to complete or quasi-complete separation, as indicated by extremely large standard errors and non-informative *p*-values. Logistic regression was therefore deemed not feasible for this dataset, as pre-specified. Missing proportions were instead compared descriptively between randomized groups at each assessment time point. In the intervention group, proportions of missing outcome data were 45.5% at T1, 72.7% at T2, 72.7% at T3, and 36.4% at T4; corresponding proportions in the control group were 41.7%, 50.0%, 83.3%, and 41.7%, respectively. These figures indicate that missing proportions were broadly comparable between groups across most time points, with no systematic pattern of differential missingness favoring either group, although the small number of participants at each assessment precludes any formal conclusions regarding the missing-data mechanism.

### 3.3. Longitudinal Descriptive Results for Patient-Reported Outcomes

Longitudinal descriptive summaries of patient-reported outcomes across follow-up assessments are reported in Table 2. For the main HRQoL outcomes, PCS12 values were similar between groups at baseline and showed variable trajectories over time. In the intervention group, median PCS12 values tended to increase at intermediate follow-up assessments and remained numerically higher than in the control group at most post-baseline time points, particularly at T3 and T4. MCS12 values were also broadly comparable at baseline and showed modest fluctuations over time in both groups, without a clear or consistent between-group separation.

Health engagement scores were similar between groups at baseline and remained relatively stable throughout follow-up, with medians generally ranging between 5 and 6 in both groups. Self-rated life satisfaction was high at baseline in both groups and showed some variability over time, with numerically lower values in the control group at several post-baseline assessments, particularly at T3 and T4.

For healthcare needs, baseline total scores were somewhat higher in the intervention group than in the control group. Over follow-up, healthcare needs total scores tended to decrease in the intervention group, whereas the control group showed more variable values and numerically higher scores at later assessments. A similar descriptive pattern was observed across the domain-specific healthcare needs scores, especially for clinical support, emotional support, and education-related needs, although estimates at later time points should be interpreted cautiously because of the small number of available observations.

Exploratory between-group comparisons at individual time points, based on Mann–Whitney U tests, did not show a consistent pattern of statistically significant differences across outcomes. For PCS12, the between-group difference approached nominal significance at T4 (*p* = 0.055), whereas *p*-values for the remaining outcomes were generally larger. All between-group comparisons at individual time points are presented for descriptive purposes only, without correction for multiple comparisons, and should not be interpreted as evidence of a treatment effect.

### 3.4. Exploratory Longitudinal Mixed-Effects Analyses of PCS12 and MCS12

**Figure 3:**
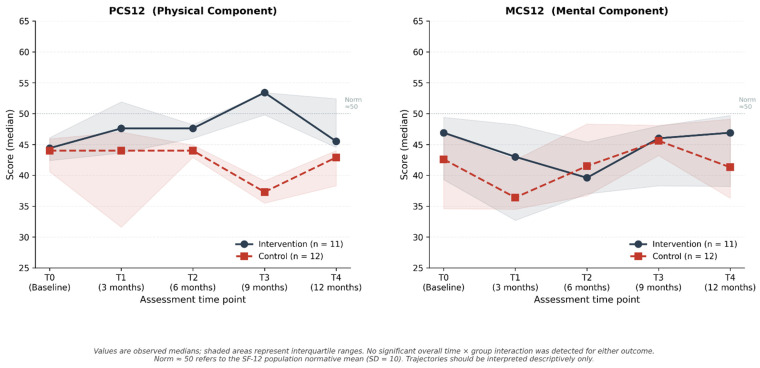
Longitudinal trajectories of PCS12 and MCS12 scores by randomized group over 12 months of follow-up. Note: Nominal time-specific contrasts are not highlighted in the figure to avoid overemphasizing exploratory differences in this small pilot dataset; no significant overall time-by-group interaction was detected for either outcome (PCS12: F(4,64) = 1.85, *p* = 0.13; MCS12: F(4,41.1) = 1.06, *p* = 0.39). The trajectories should therefore be interpreted descriptively only.

Longitudinal trajectories of PCS12 and MCS12 scores by randomized group over 12 months of follow-up are depicted in Figure 3. The figure displays observed median values and interquartile ranges at each time point, whereas inferential results are derived from exploratory linear mixed-effects models including fixed effects for time, randomized group, and their interaction, with a participant-specific random intercept. These models were fitted to examine whether longitudinal trajectories differed between groups over follow-up while accounting for within-subject correlation and incomplete repeated measurements. In a small pilot dataset with sparse repeated measurements and no correction for multiple comparisons, isolated nominal *p*-values of this type are highly vulnerable to random variation and cannot be considered even weak evidence of a treatment effect.

For PCS12, the time-by-group interaction was not statistically significant (F(4,64) = 1.85, *p* = 0.13), suggesting no clear evidence of differential trajectories across the follow-up period. Formally, a non-significant global time-by-group interaction implies that all subsequent time-specific pairwise comparisons are nominal, regardless of their individual *p*-values, and cannot be considered even weak evidence of a treatment effect. For completeness, exploratory model-based contrasts showed nominally higher PCS12 values in the intervention group at selected post-baseline time points: T1 (mean difference = 8.20, 95% CI 0.80 to 15.61, *p* = 0.030), T3 (mean difference = 13.68, 95% CI 1.17 to 26.19, *p* = 0.033), and T4 (mean difference = 7.45, 95% CI 0.35 to 14.55, *p* = 0.040). No adjustment for multiple comparisons was applied to these time-specific contrasts; given the absence of a statistically significant global interaction, the pilot sample size, and the exploratory nature of the analyses, these findings should be interpreted as hypothesis-generating only and not as evidence of treatment efficacy. Model fit indices for the PCS12 model are reported for descriptive purposes (AIC = 436.8, BIC = 462.7). The variance components extracted from the model confirmed the absence of meaningful between-subject heterogeneity in PCS12 over the observed follow-up: the estimated between-subject variance was τ^2^ = 0.00 and the residual variance was σ^2^ = 43.90. The corresponding singular-fit warning indicates that the random-intercept variance was estimated at the boundary of its parameter space. Residual diagnostics also suggested limited model robustness, further reinforcing the descriptive and hypothesis-generating status of these estimates.

For MCS12, the mixed-effects model showed no statistically significant time-by-group interaction (F(4,41.1) = 1.06, *p* = 0.39), and model-based pairwise contrasts did not identify statistically significant between-group differences at any follow-up assessment. Model fit indices are reported descriptively (AIC = 447.9, BIC = 473.8). The variance components extracted from the model indicated moderate between-subject stability of MCS12 across follow-up: the estimated between-subject variance was τ^2^ = 35.76, and the residual variance was σ^2^ = 32.80. These values are consistent with a moderate degree of within-subject coherence and with the originally reported ICC of 0.56 for MCS12. However, the reduced availability of MCS12 observations at later time points limits the precision and stability of these estimates. Residual diagnostics did not reveal any major departures from model assumptions, but their sensitivity is limited in a dataset of this size.

### 3.5. Within-Subject Correlations and Intraclass Correlation Coefficients

**Figure 4:**
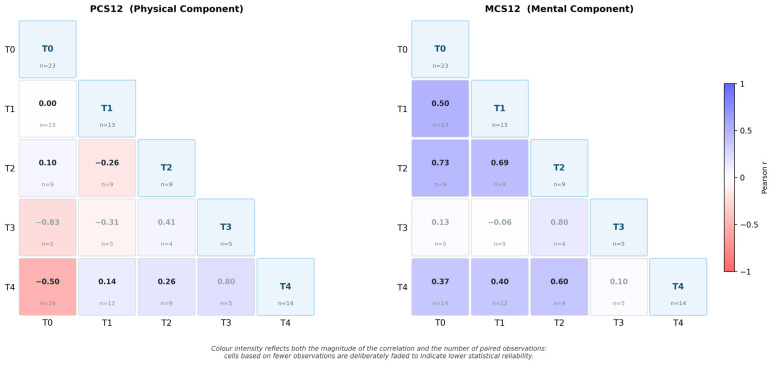
Within-subject Pearson correlation matrices for PCS12 and MCS12 across five follow-up assessment time points (T0–T4). Note: Each cell reports the Pearson correlation coefficient and the number of paired observations contributing to that estimate. Colour intensity reflects both the magnitude of the correlation and the number of paired observations, so that coefficients based on fewer observations are deliberately displayed with lower visual intensity. Several coefficients were based on very small paired samples (minimum n = 4 at T2–T3 and n = 5 at T3–T4); at these sample sizes, correlation estimates are highly unstable and confidence intervals are expected to be very wide. Significance markers, where shown, are retained for descriptive completeness only and should not be interpreted as evidence of statistically reliable associations. All estimates should be regarded as descriptive and hypothesis-generating only.

Within-subject Pearson correlation matrices for PCS12 and MCS12 across the five assessment time points are displayed in Figure 4.

For PCS12, the correlation structure was heterogeneous and lacked consistent directionality (Figure 4, left panel). Adjacent time-point correlations were near zero or weakly positive for early follow-up (T0–T1: r = 0.00, n = 13; T0–T2: r = 0.10, n = 9), whereas correlations involving T3 were strongly negative (T0–T3: r = −0.83, n = 5; T1–T3: r = −0.31, n = 5), and the T3–T4 correlation was strongly positive (r = 0.80, n = 5). These values are retained for transparency but should be interpreted as descriptive only. This erratic pattern, likely driven by the very small number of observations at T3, is consistent with the near-zero ICC for PCS12 (ICC ≈ 0.00), suggesting that between-subject variability appeared small relative to within-subject fluctuation in this sample. This estimate should, however, be interpreted with considerable caution: it is based on a very small number of paired observations at several time points, and may partly reflect numerical instability arising from sparse longitudinal sampling rather than a true measurement property of PCS12.

For MCS12, the correlation structure was substantially more coherent and predominantly positive across time point pairs with sufficient data (Figure 4, right panel). Moderate-to-high positive correlations were observed between T0 and T1 (r = 0.50, n = 13), T0 and T2 (r = 0.73, n = 9), T1 and T2 (r = 0.69, n = 9), and T2 and T3 (r = 0.80, n = 4). Because several coefficients were based on very small paired samples, these estimates are retained for transparency but should not be interpreted inferentially. *p*-values for individual correlation coefficients are not reported in the text, given the extremely small numbers of paired observations at several time points; their inclusion would create a false impression of inferential precision. All estimates should be regarded as descriptive and highly unstable (see Figure 4 note). This pattern is consistent with the moderate ICC for MCS12 (ICC = 0.56), indicating that approximately 56% of the total variance in mental health–related quality of life was attributable to stable between-subject differences rather than within-subject fluctuation over time.

### 3.6. Implications for Future Trial Planning: Variability, Retention, and Sample Size Scenarios

Empirical estimates derived from the pilot dataset were used to inform planning scenarios for a future confirmatory randomized controlled trial. At the final follow-up assessment (T4), the observed standard deviation of PCS12 was 8.6 among participants with available data (n = 14). However, because this estimate was derived from a small number of participants, it should not be treated as a fixed design constant. The approximate 95% confidence interval for the true PCS12 standard deviation ranged from 6.3 to 13.5, indicating substantial uncertainty around the variability estimate. The mean change in PCS12 from baseline to T4 was −0.9, with a standard deviation of 10.9 (n = 14). Retention at T4 was 60.9% overall, corresponding to 14 of 23 randomized participants with available outcome data at the final assessment.

Using the point estimate of SD = 8.6, a future two-arm parallel-group trial with two-sided α = 0.05, 80% power, and an assumed clinically relevant between-group difference of 5 PCS12 points would require approximately 47 participants per group under a cross-sectional PCS12-at-T4 endpoint scenario. After inflation for the observed T4 retention rate of 60.9%, this corresponds to approximately 77 randomized participants per group. However, sensitivity analyses based on the lower and upper confidence limits of the SD showed a wide range of possible requirements, from approximately 25 to 115 analyzable participants per group, corresponding to approximately 41 to 189 randomized participants per group after attrition inflation. These values highlight that the observed point estimate is useful for preliminary planning but should be validated in a larger multicenter dataset before being adopted as a definitive sample size assumption.

## 4. Discussion

This single-center pilot randomized trial was designed to assess the feasibility of repeated PRO collection and to generate empirical parameters for planning a future, adequately powered confirmatory trial of structured transition care in adolescents with CHD. Consistent with current methodological guidance for pilot and feasibility randomized trials [23,35], the primary focus was not on intervention efficacy (for which the sample was deliberately underpowered) but on feasibility indicators, retention dynamics, missingness patterns, outcome variability, and the longitudinal correlation structure of the two main HRQoL measures. Overall, longitudinal PRO collection was feasible in this real-world clinical setting, but the pilot also highlighted meaningful methodological challenges, including incomplete intermediate follow-up, variable retention, and limited precision of longitudinal effect estimates. At the same time, the study yielded useful information on outcome variability, within-subject dependence, and the divergent behavior of PCS12 and MCS12, which are findings that have direct implications for endpoint selection and analytic strategy in the planned confirmatory trial.

### 4.1. Feasibility and Retention: What the Pilot Actually Shows

More than half of randomized participants in both groups contributed outcome data at the final 12-month assessment (63.6% in the intervention; 58.3% in the control), but retention declined substantially at intermediate time points and followed a clearly non-monotonic pattern across follow-up. This observation is methodologically important and more nuanced than a simple attrition statistic [36,37]. It indicates that, in this population and setting, later follow-up is achievable, but that repeated assessments at shorter intervals are vulnerable to intermittent non-response and variable engagement. Importantly, the very low completion rates at T2 and T3 have direct methodological consequences beyond simple attrition: they reduce the number of paired observations available for estimating within-subject correlations and ICC at those time points, and may contribute to the instability of correlation estimates observed in the PCS12 matrix. This study should therefore be understood as demonstrating the feasibility of partial longitudinal data collection, not the feasibility of stable, complete repeated measurements across all five planned assessments. The pilot, therefore, suggests that future trials should incorporate explicit retention-support strategies and plan for incomplete repeated measurements even when the final endpoint remains reachable for a substantial proportion of participants [38].

The observed 12-month retention is broadly consistent with the attrition patterns reported in comparable transition-care trials in CHD, where follow-up completion rarely exceeds 70–80% over a 12- to 18-month horizon [39,40,41,42,43,44]. In a recent multicenter trial that enrolled 200 adolescents and young adults across five French centers [6], retention at 12 months was supported by a well-resourced multicenter infrastructure, yet still required sample size inflation to accommodate losses. Our figures, while lower, derive from a pragmatic single-center pilot rather than from a protected research infrastructure and are therefore particularly informative for realistic planning. The non-monotonic pattern, with lower completion at intermediate assessments (T2–T3) and partial recovery at T4, suggests that intermediate losses may reflect logistical factors (school schedules, holidays, competing adolescent commitments) rather than genuine disengagement, and supports the notion that a learner, but a strategically placed assessment schedule, may be more efficient than five closely spaced time points.

### 4.2. Missing Data and the Plausibility of the Missing-at-Random Assumption

Missing data were limited overall and concentrated in longitudinal PROs, with no missing values for baseline socio-demographic or clinical variables. No evidence emerged of differential missingness between randomized groups, and baseline PCS12 and MCS12 values did not differ between participants with and without missing follow-up data. This pattern did not identify obvious differential attrition by group, but does not constitute evidence in support of the missing-at-random assumption; missing-at-random was accordingly treated as a working analytical assumption only, as is standard practice in pilot longitudinal studies where formal verification is not feasible [45]. Its plausibility cannot be formally established from these data, because baseline comparisons cannot rule out dependence of later missingness on intermediate PRO values or on external variables not captured in the dataset. The pre-specified logistic regression model was deemed not feasible due to complete or quasi-complete separation and was replaced, as pre-specified, by a descriptive comparison of missing proportions between groups. The principal contribution of the missing-data analyses is therefore not that they establish the missing-data mechanism, but that they do not reveal an obvious pattern of differential attrition by group in this pilot sample.

### 4.3. Divergent Measurement Properties of PCS12 and MCS12

One of the most consequential findings of this pilot is the marked divergence in the variance structure of the two primary HRQoL outcomes. The near-zero ICC for PCS12 suggests that, in this pilot sample, between-subject differences were small relative to within-subject variability over time. However, as this estimate is based on sparse repeated observations, particularly at T3 where n = 5, it may partly reflect sampling instability rather than a stable property of the measure, and should not be treated as a definitive characterization of PCS12 longitudinal behavior in this population. By contrast, the ICC for MCS12 was 0.56, indicating that more than half of the total variance in mental HRQoL was attributable to stable between-subject differences. The correlation matrices reinforced this distinction: PCS12 showed heterogeneous, unstable correlations over time, whereas MCS12 showed more coherent, positive correlations across several time-point pairs.

These estimates stand in contrast with published test–retest reliability coefficients for the SF-12 in adult populations, where ICCs typically exceed 0.70 for PCS and range from 0.55 to 0.65 for MCS [46,47]. The discrepancy likely reflects two converging factors: the developmental variability in physical functioning and symptom perception during adolescence, a period of rapid somatic, hormonal, and social change, and the dynamic interaction between CHD-specific symptoms and the evolving demands of daily life during transition [48,49,50,51]. Mental HRQoL, by contrast, appears to reflect more trait-like psychological characteristics that persist across adolescence, consistent with longitudinal HRQoL research in adolescents with chronic conditions [52].

These observations should, however, be interpreted cautiously: several correlation coefficients were based on very small numbers of paired observations, and the ICC estimates themselves carry substantial uncertainty in a pilot of this size. They should inform planning, but not be treated as fixed properties of the measures. Within these limits, the findings suggest that PCS12 and MCS12 captured different longitudinal properties in this sample (i.e., PCS12 dominated by within-individual fluctuation, MCS12 influenced by stable between-subject differences) with direct consequences for analytic efficiency: repeated PCS12 measurements may not yield the efficiency gain expected when within-subject correlation is moderate or high, whereas repeated MCS12 measurements may contain substantial within-individual redundancy.

### 4.4. Exploratory Longitudinal Signals: Interpretation Within a Pilot Framework

For PCS12, the global time-by-group interaction was not statistically significant, yet exploratory model-based contrasts suggested higher values in the intervention group at T1, T3, and T4. In a pilot context, these nominal contrasts should not be interpreted as indicators of treatment responsiveness or as weak evidence of a treatment effect. In a small pilot dataset with sparse repeated measurements and no correction for multiple testing, isolated nominally significant *p*-values following a non-significant global interaction test are highly vulnerable to random variation and carry no inferential weight. They are reported here solely as descriptive, hypothesis-generating observations to be tested in a future adequately powered confirmatory trial. The direction and relative magnitude of these contrasts are compatible with the hypothesis that the intervention may influence PCS12, but this possibility requires evaluation in an adequately powered confirmatory trial.

For MCS12, the pattern was qualitatively different. Neither the global interaction test nor time-specific contrasts suggested meaningful between-group separation, and descriptively, MCS12 fluctuated over time in both groups without a consistent pattern favoring one arm. This pattern is also consistent with prior literature indicating that transition interventions tend to show their most immediate effects on knowledge, self-management, and physical functioning, whereas effects on mental HRQoL are more distal and may require longer follow-up or interventions that are more explicitly targeting psychological domains [52].

### 4.5. Implications for the Design of the Future Confirmatory Trial

An important implication of the pilot concerns endpoint definition. In the observed data, the cross-sectional variability of PCS12 at T4 (SD = 8.6) was lower than that of baseline-to-T4 change scores (SD = 10.9), and the corresponding sample size was materially smaller for a cross-sectional endpoint at T4 than for a change-score endpoint. This does not prove that a cross-sectional endpoint is universally superior (such a conclusion would depend on assumptions about baseline adjustment, measurement reliability, and missing-data handling), but within this dataset, the cross-sectional PCS12-at-T4 scenario was associated with a lower estimated sample size than the change-score scenario. Given that only 14 participants contributed T4 primary outcome data, this finding should be interpreted as a provisional planning consideration rather than as a definitive endpoint-selection recommendation. These planning recommendations are necessarily derived from a single tertiary center and should be validated across multiple sites before being adopted as fixed design constants for the confirmatory trial.

Three provisional design considerations emerge. First, PCS12 at 12 months, analyzed cross-sectionally using ANCOVA adjusted for baseline, may be considered among the endpoint formulations to be evaluated further, with linear mixed-effects models prespecified as supportive or sensitivity analyses under a missing-at-random framework, rather than assumed to guarantee efficiency by themselves [53]. Based on the observed PCS12 variability at T4 (SD = 8.6) and assuming a between-group difference of 5 points, i.e., consistent with published minimally important differences for SF-12 scores [54], approximately 47 participants per group would be required with two-sided α = 0.05 and 80% power, inflated to approximately 77 per group to accommodate the observed retention of ~61%. However, because the SD estimate was derived from only 14 participants, this point estimate varied widely depending on the true variability: sensitivity scenarios based on the 95% confidence interval for the SD ranged from approximately 25 to 115 analyzable participants per group, corresponding to approximately 41 to 189 randomized participants per group after attrition inflation. Therefore, the point estimate should be interpreted as a preliminary planning anchor rather than a definitive sample size requirement. Second, retention efforts should focus not only on preventing dropout altogether, but on improving the consistency of participation across intermediate follow-up assessments; structured reminder systems, flexible contact windows, and tighter monitoring of follow-up completion are feasibility priorities, not merely operational details [55,56]. Third, the assessment schedule could be streamlined without substantial loss of information, given the instability of PCS12 over time and the within-subject redundancy of MCS12. Taken together, these recommendations support a trial design in which the final follow-up assessment remains central, baseline values are adjusted analytically, and longitudinal models serve a supportive rather than primary analytic role.

### 4.6. Strengths and Limitations

The main strengths of this pilot study are the pragmatic single-center design in a real-world clinical setting, the use of validated PROs with established psychometric properties, transparent reporting of feasibility and methodological parameters consistent with CONSORT guidance for pilot trials [25], and the explicit derivation of data-driven sample size scenarios that translate directly into planning decisions.

Several limitations must be acknowledged. First, the sample size (n = 23) was very small, which limits the precision of all estimates, including correlation coefficients, ICCs, mixed-model coefficients, and sample size parameters. Specifically, the ICC estimates themselves carry substantial uncertainty that is not captured by point estimates alone: given the small sample size and the number of available repeated observations, confidence intervals for the ICC of both PCS12 and MCS12 would be expected to be very wide and unstable, and the point estimates should not be treated as stable or precise characterizations of the within-subject correlation structure. We therefore refrained from calculating confidence intervals for the ICCs because the sample size and number of repeated observations were insufficient to produce stable interval estimates. Reporting confidence intervals for ICCs in the future confirmatory trial, where the sample size will be substantially larger, will be essential for meaningful interpretation.

Second, the single-center design conducted within a tertiary pediatric referral center limits the generalizability of the findings in several respects. Patients enrolled at Meyer Children’s Hospital represent a specific clinical context characterized by dedicated pediatric cardiology expertise, structured follow-up programs, and proximity to specialized transition resources, which may differ substantially from community hospitals, secondary care centers, or settings with less developed transition infrastructure. Furthermore, the case-mix of a tertiary referral center, including a higher proportion of moderate-to-complex CHD diagnoses and more engaged clinical teams, may not fully reflect the broader population of adolescents with CHD across different healthcare systems or countries. The retention patterns, missingness structure, and variance estimates derived from this sample should therefore be interpreted as context-specific, and their transferability to other settings cannot be assumed. These limitations will be addressed in the future confirmatory trial, which is designed as a multicenter study to capture between-center variability and improve the representativeness of the enrolled sample.

Third, several analyses relied on sparse data at later follow-up points, especially at T3, rendering some descriptive patterns unstable. Relatedly, the linear mixed-effects models were fitted with a relatively large number of fixed-effect parameters, i.e., five time points, one group indicator, and four interaction terms, relative to the available sample size of 23 participants. This parameterization may have resulted in model instability, particularly for MCS12, where fewer observations were available at later time points. The variance component estimates, model-based contrasts, and associated confidence intervals should therefore be interpreted with caution as potentially imprecise and are reported primarily to generate planning hypotheses rather than to characterize the longitudinal outcome structure definitively.

Fourth, although mixed-effects models allow inclusion of incomplete repeated measurements, the plausibility of the missing-at-random assumption cannot be fully verified from these data and remains only partially supported.

Fifth, allocation concealment was based on a prespecified center-specific randomization list applied sequentially by enrollment order, which does not constitute adequate concealment: with permuted blocks of size 4, the enrolling investigator could potentially foresee upcoming group assignments toward the end of each block. This introduces a risk of selection bias, which is the possibility that awareness of imminent assignments may have influenced eligibility assessment or enrollment decisions, and should be considered when interpreting the baseline comparability of the two groups. This limitation will be addressed in the future confirmatory trial through a centralized web-based randomization platform, which will ensure allocation concealment independent of the enrolling investigator.

Sixth, because the pilot was not powered for efficacy testing, any between-group differences observed in the exploratory analyses should be interpreted cautiously and not treated as confirmatory. Additional PROs collected in the pilot were included primarily for descriptive and feasibility purposes in the present report.

Seventh, the 3-month assessment schedule, while selected to capture longitudinal outcome trajectories across the transition period and consistent with the parent TELEMACO protocol, may itself have contributed to participant burden and intermittent non-response, particularly at T2 and T3. Although the non-monotonic completion pattern observed at intermediate time points may partly reflect logistical factors inherent to adolescence, such as school schedules and competing commitments, it cannot be excluded that the frequency of assessments itself was a contributing factor. Future trials should evaluate whether a reduced number of strategically placed assessments, prioritizing the final endpoint, would maintain adequate statistical efficiency while reducing participant burden and improving completion at intermediate time points.

Finally, although no intervention-related adverse events were identified in the available study documentation, harms and unintended effects were not collected through a dedicated standardized reporting form. This limits the ability to formally evaluate the safety and acceptability profile of the transition intervention, including potential burden or psychological distress related to repeated assessments. Future confirmatory trials should include prospective monitoring of adverse events and unintended consequences alongside feasibility and effectiveness outcomes.

## 5. Conclusions

This single-center pilot randomized trial demonstrated that repeated collection of patient-reported outcomes in adolescents with CHD is feasible within a structured transition care setting, although follow-up completion was inconsistent across intermediate assessments and only partially recovered at 12 months. The main contribution of this pilot is the empirical foundation that the results provide for designing, analyzing, and operationalizing a future multicenter trial of structured transition care in adolescents with congenital heart disease. The study was not designed to evaluate intervention efficacy, and its findings should not be interpreted in that light; rather, it generated clinically and methodologically relevant information to inform the design of a future adequately powered confirmatory trial. Specifically, the pilot produced empirical estimates of retention, missing-data patterns, longitudinal outcome variability, and within-subject dependence, and provided preliminary evidence that PCS12 and MCS12 may differ substantially in their longitudinal measurement properties in this population. These findings support the feasibility of a future confirmatory multicenter trial but also indicate that its design should explicitly account for incomplete repeated measurements, prioritize retention at the final endpoint rather than at all intermediate assessments, and ground endpoint selection in the empirically observed variance structure rather than on assumptions. Within this pilot dataset, PCS12 measured at 12 months may be considered a provisional endpoint option for further evaluation; a baseline-adjusted cross-sectional formulation was associated with a lower estimated sample size than a change-score approach in the observed data. Given the small sample size, intermittent missingness, and sparse repeated observations at later assessments, these observations should be regarded as exploratory planning signals only and interpreted with caution. In particular, the sample-size point estimate varied widely depending on the true PCS12 variability, from approximately 25 to 115 analyzable participants per group, and should not be considered as a fixed design requirement.

## Figures and Tables

**Table 1:** Baseline characteristics of the randomized sample.

Characteristic	Intervention Group (N = 11)	Control Group (N = 12)
Sex, n (%)		
Male	6 (54.5)	3 (25.0)
Female	5 (45.5)	9 (75.0)
Age, years	14.0 (13.0–15.5)	14.0 (12.8–14.2)
Marital status, n (%)		
Unmarried	11 (100.0)	12 (100.0)
Education, n (%)		
Primary school diploma	9 (81.8)	11 (91.7)
High school diploma	2 (18.2)	1 (8.3)
Employment, n (%)		
Student status	11 (100.0)	12 (100.0)
Body mass index, kg/m^2^	20.1 (18.0–21.6)	18.7 (18.1–20.9)
CHD complexity, n (%)		
Moderate CHD	8 (72.7)	10 (83.3)
Complex CHD	3 (27.3)	2 (16.7)
Comorbidity burden, n (%)		
1 Co-morbidity	3 (27.3)	1 (8.3)
2 Co-morbidities	0 (0.0)	0 (0.0)
≥3 Co-morbidities	0 (0.0)	1 (8.3)
NYHA class, n (%)		
Class I	11 (100.0)	11 (91.7)
Class II	0 (0.0)	1 (8.3)
PCS12	44.4 (42.4–46.1)	44.0 (40.6–45.9)
MCS12	46.9 (39.3–49.4)	42.6 (34.6–46.8)
Health engagement	6.0 (5.0–7.0)	6.0 (5.0–7.0)
Life satisfaction VAS	85.0 (72.5–95.0)	87.5 (75.0–91.2)
Healthcare needs total	76.8 (66.1–80.4)	65.2 (53.1–74.1)

Note: Continuous variables are reported as median (IQR); categorical variables as n (%). Percentages are calculated within randomized group. No baseline data were missing. Baseline summaries are descriptive only.

**Table 2:** Longitudinal descriptive statistics of patient-reported outcomes by study group.

Outcome/ Time Point	Intervention Group (N = 11) Median (Q1–Q3)	Control Group (N = 12) Median (Q1–Q3)	*p*-Value
**PCS12**			
T0	44.4 (42.4–46.1)	44.0 (40.6–45.9)	0.441
T1	47.6 (43.6–51.9)	44.0 (31.6–47.0)	0.520
T2	47.6 (46.0–48.2)	44.0 (42.9–44.9)	0.245
T3	53.4 (49.8–53.4)	37.3 (35.5–39.1)	0.139
T4	45.5 (44.5–52.4)	42.9 (38.3–44.0)	0.055
**MCS12**			
T0	46.9 (39.3–49.4)	42.6 (34.6–46.8)	0.406
T1	43.0 (32.7–48.2)	36.4 (34.5–42.5)	0.721
T2	39.6 (37.0–45.4)	41.5 (36.7–48.3)	1.000
T3	46.0 (38.3–48.0)	45.6 (43.2–48.1)	0.773
T4	46.9 (38.2–49.7)	41.3 (36.3–49.1)	0.949
**Health engagement**			
T0	6.0 (5.0–7.0)	6.0 (5.0–7.0)	0.974
T1	6.0 (5.5–6.0)	5.5 (5.0–6.0)	0.838
T2	6.0 (6.0–6.0)	6.0 (5.0–7.0)	1.000
T3	5.0 (5.0–5.0)	4.5 (4.2–4.8)	0.617
T4	6.0 (5.0–6.0)	5.5 (5.0–6.8)	1.000
**Life satisfaction VAS**			
T0	85.0 (72.5–95.0)	87.5 (75.0–91.2)	0.780
T1	85.0 (75.0–95.0)	77.5 (56.2–81.5)	0.232
T2	81.0 (73.0–88.0)	77.5 (70.0–92.5)	1.000
T3	80.0 (75.0–85.0)	67.5 (58.8–76.2)	0.699
T4	84.5 (81.0–92.5)	70.0 (50.0–90.0)	0.280
**Healthcare needs total**			
T0	76.8 (66.1–80.4)	65.2 (53.1–74.1)	0.102
T1	66.1 (57.1–71.4)	78.6 (70.5–85.3)	0.170
T2	55.4 (50.0–67.0)	69.6 (51.3–81.2)	0.604
T3	50.0 (40.2–59.8)	90.2 (87.1–93.3)	0.245
T4	58.9 (44.6–74.1)	83.9 (67.0–87.5)	0.481
**Clinical support**			
T0	75.0 (58.3–83.3)	66.7 (47.9–77.1)	0.420
T1	66.7 (50.0–66.7)	83.3 (77.1–95.8)	0.165
T2	66.7 (58.3–79.2)	70.8 (39.6–89.6)	1.000
T3	54.2 (52.1–56.2)	91.7 (87.5–95.8)	0.245
T4	50.0 (33.3–75.0)	91.7 (66.7–95.8)	0.364
**Emotional support**			
T0	91.7 (70.8–95.8)	75.0 (58.3–85.4)	0.143
T1	66.7 (58.3–75.0)	83.3 (68.8–91.7)	0.405
T2	75.0 (58.3–79.2)	75.0 (56.2–93.8)	0.895
T3	50.0 (37.5–62.5)	87.5 (85.4–89.6)	0.245
T4	58.3 (45.8–79.2)	83.3 (62.5–87.5)	0.521
**Continuum of care**			
T0	58.3 (50.0–75.0)	50.0 (41.7–52.1)	0.060
T1	41.7 (41.7–50.0)	58.3 (58.3–58.3)	0.299
T2	33.3 (16.7–45.8)	45.8 (27.1–70.8)	0.601
T3	75.0 (75.0–75.0)	79.2 (72.9–85.4)	1.000
T4	50.0 (37.5–75.0)	75.0 (50.0–87.5)	0.520
**Healthcare education**			
T0	80.0 (77.5–87.5)	72.5 (58.8–86.2)	0.154
T1	70.0 (70.0–75.0)	82.5 (71.2–93.8)	0.356
T2	70.0 (60.0–75.0)	72.5 (66.2–86.2)	0.795
T3	62.5 (58.8–66.2)	97.5 (96.2–98.8)	0.245
T4	70.0 (52.5–80.0)	90.0 (67.5–92.5)	0.369

Note: Values are reported as median (Q1–Q3). Between-group comparisons at each time point were performed using Mann–Whitney U tests and are presented for descriptive purposes only. No adjustment for multiple comparisons was applied. Later follow-up estimates, particularly at T2–T4, should be interpreted cautiously because of the limited number of available observations.

## Data Availability

The anonymized data supporting the findings of this study are available in the Supplementary Materials.

## Supplementary Materials

The following supporting information can be downloaded at: https://www.mdpi.com/article/10.3390/children13060742/s1, Table S1: CONSORT checklist; Table S2: Anonymized data supporting the findings of this study.

## Abbreviations

The following abbreviations are used in this manuscript:

**Abbreviation**	**Full Term**
CHD	Congenital Heart Disease
PRO	Patient-Reported Outcome
PCS12	Physical Component Summary (12-Item Short Form Health Survey)
MCS12	Mental Component Summary (12-Item Short Form Health Survey)
HRQoL	Health-Related Quality of Life
ICC	Intraclass Correlation Coefficient
NYHA	New York Heart Association
VAS	Visual Analogue Scale
PHE	Patient Health Engagement
I-HNS-CHD-s	Italian Healthcare Needs Scale for Youth with Congenital Heart Disease
RCT	Randomized Controlled Trial
CONSORT	Consolidated Standards of Reporting Trials
T0–T4	Time Points (Baseline to 12-Month Follow-Up Assessments)
