# Supplementary material for: Feasibility of Repeated Patient-Reported Outcome Collection and Trial Design Implications for Structured Transition Care in Adolescents with Congenital Heart Disease: A Single-Center Pilot Randomized Controlled Study
Source: Children (Basel). 2026 May 26;13(6):742. doi: 10.3390/children13060742 (PMC13297527; doi:10.3390/children13060742)
Supplement: Supplementary file 1 [file children-13-00742-s001.zip › children-4331584-supplementary/Supplementary File 1.pdf]

Table S1. *CONSORT checklist*

Section / Topic	No	Reported on page no.
Title and structured abstract	1a	p. 1
Title and structured abstract	1b	p. 1
Trial registration	2	p. 2; final statements
Protocol and statistical analysis plan	3	methods
Data sharing	4	Final statements / Supplementary Materials
Funding and role of funders	5a	Final statements
Conflicts of interest	5b	Final statements
Background and rationale	6	pp. 1–2
Objectives	7	p. 2
Patient/public involvement	8	Not applicable
Trial design	9	p. 2
Changes to trial protocol	10	p. 4
Trial setting	11	p. 2
Eligibility criteria for participants	12a	p. 2
Eligibility criteria for sites/providers	12b	Not applicable
Intervention and comparator	13	pp. 2–3
Outcomes	14	pp. 3–4
Harms	15	Not applicable
Sample size	16a	p. 3
Interim analyses/stopping guidelines	16b	Not applicable
Sequence generation	17a	p. 4
Randomisation restrictions	17b	p. 4
Allocation concealment	18	p. 4
Implementation	19	p. 4
Blinding	20a	p. 4
Blinding method	20b	p. 4 / not applicable for participants/providers
Statistical methods	21a	pp. 4–5
Analysis population	21b	pp. 5–6
Missing data handling	21c	pp. 4–5
Additional analyses	21d	pp. 4–5
Participant flow	22a	p. 5; Figure 1
Losses/exclusions after randomisation	22b	p. 5; Figure 1
Recruitment dates/follow-up period	23a	Follow-up reported p. 2
Trial stopped/ended	23b	Not applicable
Intervention/comparator delivery	24a	pp. 2–3
Concomitant care	24b	Not reported
Baseline data	25	p. 6; Table 1
Numbers analysed, outcomes, estimation	26	pp. 6–8; Table 2; Figure 3
Harms	27	Not reported
Ancillary analyses	28	pp. 8–10; Figures 4; sample size scenarios
Interpretation	29	Discussion, pp. 10–12
Limitations	30	Discussion, p. 12
