# Supplementary material for: Feasibility of Repeated Patient-Reported Outcome Collection and Trial Design Implications for Structured Transition Care in Adolescents with Congenital Heart Disease: A Single-Center Pilot Randomized Controlled Study
Source: Children (Basel). 2026 May 26;13(6):742. doi: 10.3390/children13060742 (PMC13297527; doi:10.3390/children13060742)
Supplement: Supplementary file 1 [file children-13-00742-s001.zip › children-4331584-supplementary/Supplementary File 2.pdf]

Table S2. Anonymized data supporting the findings of this study

MCS12_TOT	PCS12_TOT	bmi	randomization	timing	participant_id
43,28	42	20	2	T0	P001
37,36	43,92	20	2	T2	P001
36,39	38,36	20	2	T4	P001
36,36	20,58	20	2	T1	P001
35,54	42,96	17,79	1	T0	P010
41,15	41,08	17,79	1	T1	P010
45,98	46,17	17,79	1	T3	P010
49,36	33,18	17,79	1	T4	P010
32,28	48,28	17,71	2	T0	P011
52,04	46,06	20,09	1	T0	P012
52,93	40,87	19,53	2	T0	P013
50,07	53,4	19	1	T3	P014
50,96	40,26	16,22	1	T0	P014
52,76	43,44	16,22	1	T1	P014
51,06	44,32	16,22	1	T2	P014
50,07	45,46	16,22	1	T4	P014
33,98	38,08	18,7	2	T0	P015
52,04	46,06	17,91	1	T0	P016
33,37	41,79	18,18	1	T0	P017
29,94	53,69	18,18	1	T1	P017
34,27	48,77	18,18	1	T2	P017
29,51	51,19	18,18	1	T4	P017
42,31	39,83	23,53	2	T0	P018
30,57	53,83	23,53	2	T1	P018
36,2	38,3	23,53	2	T4	P018
30,39	46,93	20,13	1	T0	P019
29,08	44,67	23	1	T4	P002
49,36	51,19	20	1	T1	P002
46,94	44,42	22,5	1	T0	P002
42,95	45,64	18,6	2	T0	P020
47,78	44,96	18,6	2	T1	P020
50,41	44,1	18,6	2	T2	P020
52,37	43,68	18,6	2	T4	P020
43,1	48,47	21,3	1	T0	P021
46,94	44,42	21,3	1	T4	P021
52,37	43,68	18,75	2	T0	P022
27,83	46,59	24,44	2	T0	P023
46,94	44,42	18,8	1	T0	P003
44,85	43,94	18,83	1	T1	P003
39,64	47,63	18,83	1	T2	P003
30,56	53,4	18,83	1	T3	P003
46,94	53,69	18,83	1	T4	P003
47,89	41,85	30,8	1	T0	P004
51,06	53,69	30,8	1	T4	P004
47,06	42,93	18	2	T4	P005
45,74	42,55	17,27	2	T1	P005
47,04	39,25	17,31	2	T0	P005
45,74	42,55	17,31	2	T2	P005
46,73	44,29	18,2	2	T0	P006
34,49	49,04	18,2	2	T1	P006

49,21	42,23	18,2	2	T2	P006
50,52	40,98	18,2	2	T3	P006
41,31	45,99	18,2	2	T4	P006
39,2	44,04	26	2	T1	P007
36,52	45,11	26	2	T2	P007
40,74	33,64	26	2	T3	P007
34,48	20,58	26	2	T4	P007
34,84	53,39	26,06	2	T0	P007
44,31	45,28	22	1	T0	P008
29,59	52,09	21,94	1	T1	P008
41,63	44,33	16,89	2	T0	P009
34,48	20,58	16,89	2	T1	P009
35,76	50,92	16,89	2	T2	P009
51,06	44,32	16,89	2	T4	P009
